# The Pathological Anatomy of the Human Body

**Published:** 1847-04

**Authors:** 


ART. VIII.—*The Pathological Anatomy of the Human Body.* By JULIUS VOGEL, M.D., Professor of Clinical Medicine at the University of Giessen. Translated from the German, with additions, By GEO. E. DAY, M.A., and L. M., Cantab. Member of the Royal College of Physicians; Physician to the Westein General Dispensary; Lecturer on Pathology and Animal Chemistry at the Middlesex Hospital Medical School; Member of the Pathological Society of London; and formerly senior President of the Royal Medical Society of Edinburgh. Illustrated by upwards of 100 plain and colored engravings. pp. 534, 8vo. Philadelphia: Lea and Blanchard. 1847.

If the multiplication of treatises on pathological anatomy is to be regarded as a test of the interest felt in the study, that much neglected branch of the profession is beginning to assume a position to which, in the estimation of its admirers, it has always been entitled. In the space of a few years, besides the second edition of Prof. Gross's work, we have had reprints of Andral, Hope, Hasse, and here we have a new volume the title of which is given above. The Germans and French have always taken the lead in the cultivation of pathological anatomy. Even up to this time no systematic work on the subject has been produced in England. The volume before us reaches us through the hands of Dr. Day, to whom we owe the translation of Simon's Animal Chemistry. It is a work of decided ability, learning, and research, and of a convenient size. The translator mentions as among its distinguishing merits, that it embraces the recent discoveries effected by chemistry and the microscope. These will be found to be numerous and interesting. Physiology and pathological anatomy are assuming a new aspect since the microscope and modern analysis began to be turned upon them. Vogel has availed himself of all the light afforded by these copious sources of information. We have not room to notice it at greater length, and are compelled reluctantly to omit several other notices for the same reason.

